# Association of sociodemographic factors and internet query data with pertussis infections in Shandong, China

**DOI:** 10.1017/S0950268819001924

**Published:** 2019-11-15

**Authors:** Yuzhou Zhang, Hilary Bambrick, Kerrie Mengersen, Shilu Tong, Lei Feng, Li Zhang, Guifang Liu, Aiqiang Xu, Wenbiao Hu

**Affiliations:** 1 School of Public Health and Social Work, Institute of Health and Biomedical Innovation, Queensland University of Technology, Brisbane, Queensland, Australia; 2 School of Mathematical Sciences, Queensland University of Technology, Brisbane, Queensland, Australia; 3 School of Public Health and Institute of Environment and Human Health, Anhui Medical University, Hefei, Anhui, China; 4 Shanghai Children's Medical Centre, Shanghai Jiao-Tong University, Shanghai, China; 5 Shandong Provincial Centre of Disease Control and Prevention, Jinan, China

**Keywords:** Pertussis (whooping cough), public health, surveillance

## Abstract

This study explored how internet queries vary in facilitating monitoring of pertussis, and the effects of sociodemographic characteristics on such variation by city in Shandong province, China. We collected weekly pertussis notifications, Baidu Index (BI) data and yearly sociodemographic data at the city level between 1 January 2009 and 31 December 2017. Spearman's correlation was performed for temporal risk indices, generalised linear models and regression tree models were developed to identify the hierarchical effects and the threshold between sociodemographic factors and internet query data with pertussis surveillance. The BI was correlated with pertussis notifications, with a strongly spatial variation among cities in temporal risk indices (composite temporal risk metric (CTRM) range: 0.59–1.24). The percentage of urban population (relative risk (RR): 1.05, 95% confidence interval (CI) 1.03–1.07), the proportion of highly educated population (RR: 1.27, 95% CI 1.16–1.39) and the internet access rate (RR: 1.04, 95% CI 1.02–1.05) were correlated with CTRM. Higher RRs in the three identified sociodemographic factors were associated with higher stratified CTRM. The percentage of highly educated population was the most important determinant in the BI with pertussis surveillance. The findings may lead to spatially-specific criteria to inform development of an early warning system of pertussis infections using internet query data.

## Introduction

Pertussis (also known as whooping cough or 100-day cough) is a highly infectious respiratory disease with a global substantial public health burden [1]. It is recognised as a resurgent infectious disease in several countries in the last decade, such as the USA, Japan, Australia and China [2–5]. Despite extensive immunisations, pertussis still leads to large numbers of cases annually (143 963 in 2017) [6], and childhood deaths worldwide (around 63 000 aged <5 years in 2013) [7]. In China, the pertussis vaccination program was introduced in the early 1960s. However, the reported number of pertussis cases increased by 66.2% in the last decade, even though China has a high vaccination coverage of over 99% among target population [1, 5, 8, 9]. The transition of vaccine type from whole-cell pertussis vaccine to acellular vaccine, which has a lower protection level may lead to an increase of pertussis cases [10]. Furthermore, the appearance of erythromycin-resistant *Bordetella pertussis* should be alarming in China, as this may also contribute to the rising trend in pertussis infections [11, 12].

Although traditional infectious disease surveillance has high accuracy in reporting, such surveillance can be delayed up to 2 weeks from the onset of symptoms to the notification [13]. Previous studies reported that the routine surveillance data of China are typically reported with a time lag of at least 10 days, and up to two weeks [14, 15]. This delay risks public health by inhibiting timely response with control measures [16]. To better prepare for potential outbreaks and efficiently improve response times, internet search query data is now increasingly used worldwide [17–21]. This new method based on tracking the search frequency of disease-related search queries which was triggered by the people who contract a disease. These people are likely to actively seek disease-related information on the internet [22].

The great potential of using internet search query data to improve disease surveillance in China has been explored, previous studies have shown that, generally, the Baidu Index (BI) can successfully detect disease epidemics in China [14, 23]. Moreover, previous studies have shown that internet search query data has the potential in timely tracking and even prediction of pertussis infections in several countries [24–26]. However, there is a great variation in the accuracy of predictions using internet search term data by region [27, 28]. Current understanding of potential reasons for such variation is limited. Previous studies have reported that individual-level factors, such as fear based searching and variable internet-seeking behaviours may adversely affect the accuracy of internet-based surveillance [29]. Moreover, it has been reported that the higher income and education were positively associated with diseases reporting on social media [30, 31].

In China, Sina Weibo is the most popular social media platform, with the monthly active users of 242 million [32]. This number is relatively smaller then internet search users in China. Baidu is the dominating internet search engine in the country, with 665 million monthly active users [33]. Thus, it is necessary to evaluate the effects of sociodemographic factors on internet query-based disease monitoring in China, considering the huge internet search users. However, to our knowledge, there is no published studies examined the associations of population-level sociodemographic factors and internet query data for disease monitoring in China. This study aimed to examine the relationships between sociodemographic factors and internet query in pertussis detection and make useful suggestions for developing an early warning system for pertussis in Shandong province, China.

## Methods

### Study site and data collection

**Fig. 1.**
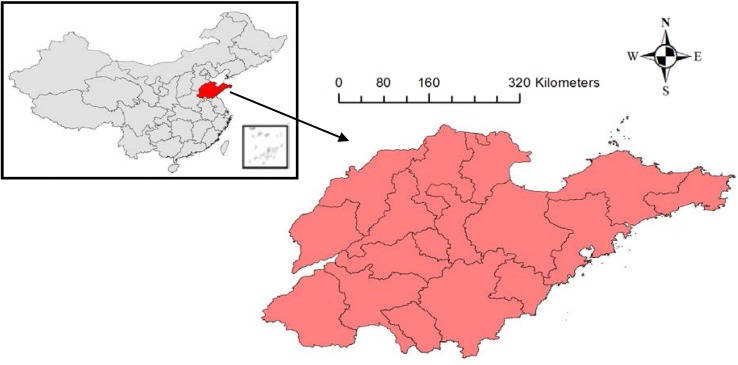
The location of Shandong province (red) in China.

Shandong province is located in the east of China (Fig. 1). It is the second largest province by population in China with nearly 100 million people and 17 cities [34]. The mean value of the population size (million) and gross domestic product (GDP) (billion yuan) at the city level were 5.79 (range: 1.35–10.31) and 394.96 (USD: 58.79) (range: 89.60–1103.73), respectively [34]. The socio-economic level is diverse across the province, ranging from extensive development in the east coastal areas to undeveloped regions in the west [35].

The weekly total numbers of cases of clinical and laboratory confirmed pertussis for each city in Shandong province were retrieved for the period of 1 January 2009 to 31 December 2017 from the Chinese National Notifiable Disease Reporting System (CNNDRS), which has been widely used in previous studies of pertussis and other infectious diseases in China [5, 36]. The detailed diagnosis confirmation of pertussis (Weisheng (WS, hygiene in Chinese) 274-2007) was issued by the Chinese Ministry of Health on 17 April 2007, and all laboratory confirmed and clinically diagnosis cases must be uploaded to CNNDRS within 24 h after diagnosis [37].

Weekly search metrics data at the city level in the same period were obtained from the BI (https://zhishu.baidu.com/). Baidu dominates the Chinese internet search market with 72% search engine market share [33]. We selected the top ten search terms (translated to English in Supplementary Table S1) which were highly correlated with the term ‘pertussis’ in the study period. The top relevant search terms were officially provided by the BI, which can be found on the BI website after searching for ‘pertussis’. Then we combined the selected search terms to one search query using ‘ + ’ in the search box of the BI to collect the search metrics data.

City-specific yearly sociodemographic data, including demographic category (total population, urban population and proportion of population at different age groups (0–14 years old, 15–64 years old and over 65 years old)), socioeconomic category (highly educated population (bachelor or higher), education years and GDP) and internet access category (internet access rate by cell phone or personal computer (PC)) were obtained from Shandong Provincial Bureau of Statistics (http://www.stats-sd.gov.cn/). These variables were selected because many of them have been reported as potential factors that may affect internet-based surveillance [38, 39].

The Office of Research Ethics and Integrity of The Queensland University of Technology provided ethical approval for this study (Approval Number: 1800000047).

### Data analysis

#### Spatial patterns of pertussis infections, search query and sociodemographic factors

As the annual incidence rate is the most common indicator in measuring the temporal risk of a disease epidemic [40], we transformed the weekly number of cases to a yearly mean incidence rate of pertussis (2009–2017) (cases/100 000 population) and then mapped the mean yearly incidence rate at the city level. To be consistent with the yearly incidence rate of pertussis in the time series, we also mapped city-specific yearly mean BI per capita (BPC) (yearly BI/total population), percentage of urban population (urban population/total population × 100), the percentage of highly educated population (highly educated population/total population × 100) and GDP per capita (GPC) (Yuan/total population), separately. Then, we mapped city-specific sociodemographic data to show the spatial variation of each factor.

#### The relationship between internet query data and pertussis infections

Four temporal risk indices were used to evaluate the relationship between pertussis surveillance and internet query (BI). First, Spearman's correlation coefficient was used to assess the overall time-series relationship between the weekly BI and pertussis notifications for each city over the study period. Second, we calculated Spearman's correlation coefficient to evaluate the association between the annual peak number of pertussis notifications (the maximum number of weekly cases) and that of the BI [41]. Third, the Spearman's correlation coefficient between increasing intensity of the BI and pertussis notifications was tested over the study period [41]. The increasing intensity index refers to the likely spreading speed during an epidemic period, which can reflect the severity of an epidemic [42], and is formulated as:
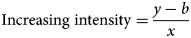
where *y* is the observed peaking pertussis notifications in an epidemic; *b* is the base level of the index, which is defined as the starting value of an epidemic and *x* is the number of weeks between the commencement and peaking week for the epidemic [41]. We also calculated this index for the BI to discover whether the BI can monitor the spread speed of pertussis in an epidemic. We used the same commencement week for the BI, but used BI's own peaking week in the analysis. An epidemic period was defined when the number of cases exceeded the median of annual weekly pertussis notifications (30).

To better evaluate the overall associations between the BI and pertussis notifications, we calculated the composite correlations for the time-series data, peaking numbers and increasing intensity. For this purpose, we separately transformed the correlation coefficients of time-series data, peaking number and increasing intensity to *Z* values using Fisher *Z*-transformation, which is widely used to compare correlation coefficients [43, 44]. Then, we calculated the average *Z* value of the three temporal risk indictors (time-series data, peaking number and increasing intensity) as the composite temporal risk metrics (CTRM). A larger value in the CTRM refers to a stronger overall association between internet query and pertussis infections, as this indicated that internet query has a composite capacity to better track the variation, the size of outbreak and the spread speed of transmission of pertussis activity.

#### Modelling the effects of sociodemographic factors on the associations between internet query and pertussis infections

A generalised linear model (GLM) was developed to fit the relationship between the composite metric and sociodemographic factors. We used the city-specific CTRM as the dependent variable and city-specific sociodemographic data as the independent variables. We assumed a log normal distribution for the response variable and a identify link to the linear model, such that:
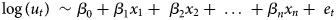
where *u*_*t*_ is the value of the CTRM; *e*_*t*_ is the error term; *β*_0_ is the intercept for the model; *x*_1_, …, *x*_*n*_ are the sociodemographic factors and *β*_1_, …, *β*_*n*_ are the corresponding regression coefficients. Multicollinearity among sociodemographic factors in each category was checked and minimised through evaluating Pearson correlations and variance inflation factors (VIF). Only one of any pair of highly-correlated factors (*r* > 0.6 or VIF > 5) in each category was included in the model [45]. The sociodemographic factors with statistically significant coefficients in the model were selected for further analysis. We calculated the relative risk (RR) with corresponding 95% confidence interval (CI) of the CTRM associated with the sociodemographic factors, relative to the smallest value the CTRM [46].

#### Stratified effects of sociodemographic factors on the relationship

To examine how the effects of identified sociodemographic factors on the relationship changed by different levels, we developed GLMs to unravel the stratified RR in the CTRM. For this purpose, we stratified the dataset into three subsets according to <30th percentile, 30–70th percentile and >70th percentile of the CTRM [47]. Then, we separately developed the GLMs for each subset to examine and compared the RRs and corresponding 95% CI of the identified sociodemographic factors.

#### Regression tree analysis

Regression tree models were developed to segment the identified sociodemographic factors into subsets that were most likely to be associated with a stronger relationship between pertussis infections and internet query data [48]. We used the city-specific Spearman's correlation coefficient of temporal risk indices as the dependent variables and sociodemographic data as the independent variables. Cross-validation was conducted to choose the best tree size by checking estimated prediction errors. The best model is defined as having the smallest tree size and an estimated error rate within one standard error of the minimum [49].

All data analyses were carried out using SPSS Statistics software, version 25 (SPSS Inc.; Chicago, IL, USA) and R package ‘MASS’, version 7.3-51.4 and ‘rpart’, version 4.1-15 [41].

## Results

### Spatial patterns of pertussis infections, internet query and sociodemographic factors

**Fig. 2.**
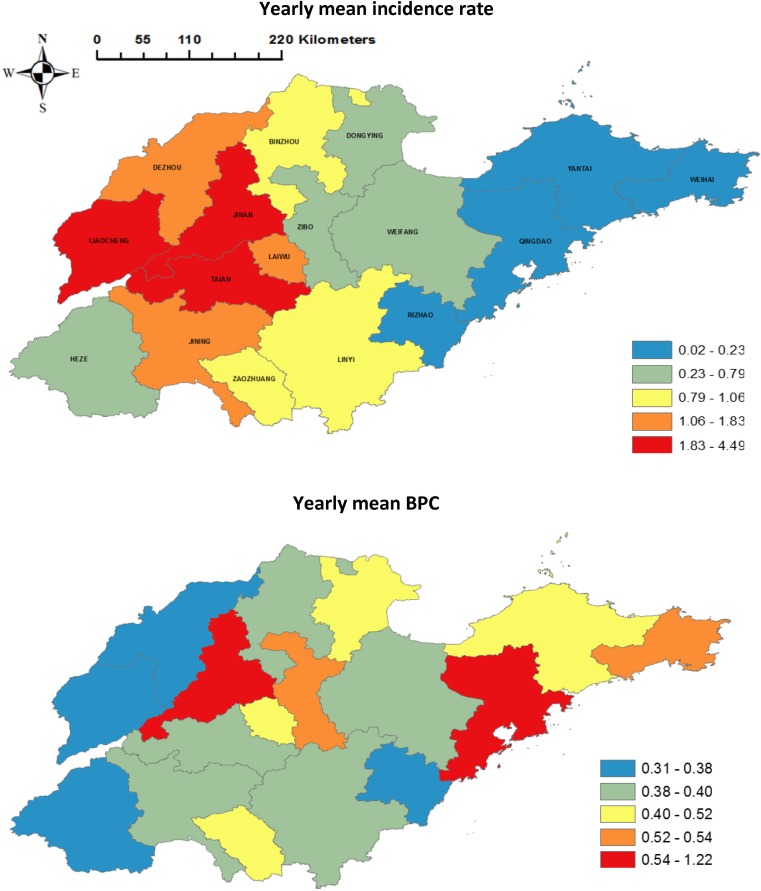
City-level yearly mean pertussis incidence rate and yearly mean BPC in Shandong province, 2009–2017.

A total of 8646 pertussis cases were reported in the province over the study period, with the largest and smallest numbers of notifications in Jinan city (2067 cases) and Qingdao city (12 cases), separately. The city-specific yearly mean pertussis incidence rate was 1.25 cases/100 000 population during the study period with great spatial variation (Fig. 2). The highest and lowest incidence rates were observed in Jinan city (4.49 cases/100 000 population) and Qingdao city (0.02 cases/100 000 population), respectively. Moreover, the city-specific yearly mean BPC was 0.49 in the province with the highest value of 1.22 in Jinan and the lowest value of 0.31 in Heze (Fig. 2). Furthermore, there were great spatial variations in sociodemographic characteristics between cities in Shandong province (Supplementary Fig. S1). Details of the city-specific sociodemographic levels in the province are shown in Table 1.

**Table 1.** The characteristics of sociodemographic factors in Shandong province by city

Sociodemographic characteristics	Mean value (minimum; maximum; standard deviation)
Percentage of urban population	49.8 (36.0; 73.4; 9.8)
Percentage of population (0–14 years)	15.0 (2.0; 20.8; 4.0)
Percentage of population (15–64 years)	74.6 (69.5; 77.9; 2.0)
Percentage of population (over 65 years)	9.9 (8.7; 12.1; 0.9)
Percentage of highly educated population	3.6 (1.1; 9.4; 2.4)
Education years	9.0 (7.9; 10.3; 0.6)
GPC (Yuan/capita)	38 486.1 (15 730.0; 87 295.0; 17 618.9)
Internet access rate (cell phone)	66.1 (51.3; 89.7; 13.6)
Internet access rate (PC)	53.1 (31.8; 88.8; 17.1)

### The association between pertussis infections and internet query

**Fig. 3.**
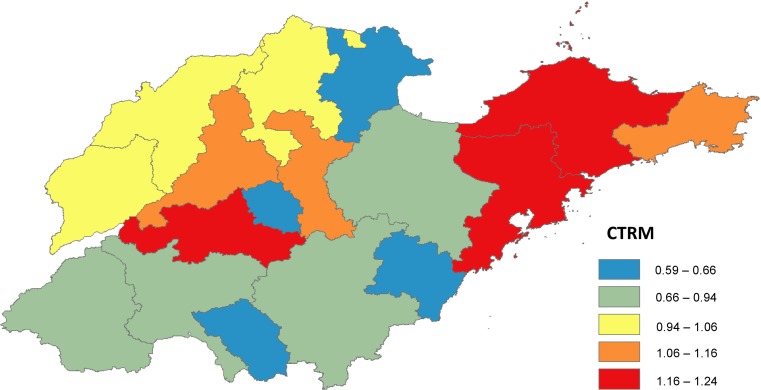
The city-specific CTRM in Shandong province, 2009–2017.

There were obvious spatial variations in the correlations over the study period. The strongest correlations between the time-series weekly pertussis notifications and BI were also found to be highest in Yantai city (correlation coefficient: 0.87; *P*-value: 0.023). Similarly, the peaks of the BI best correlated with that of pertussis activity in Qingdao city (correlation coefficient: 0.85; *P*-value: 0.012). Additionally, the highest correlation value between increasing intensity of the BI and that of pertussis epidemics was observed in Yantai (correlation coefficient: 0.83; *P*-value: 0.034) (Table 2). Then, we calculated the CTRM for each city and observed that Yantai had the highest value of 1.24 (Table 2) (Fig. 3).

**Table 2.** The correlation coefficients of temporal risk indices of pertussis infections and internet query in Shandong province by city, 2009–2017

City	Time-series	Peaking number	Increasing intensity	CTRM
Dezhou	0.68**	0.85*	0.80*	1.06
Heze	0.62**	0.69*	0.62**	0.77
Jinan	0.78*	0.85*	0.80*	1.13
Jining	0.68**	0.79*	0.68*	0.91
Laiwu	0.52**	0.55**	0.52**	0.59
Liaocheng	0.72*	0.81*	0.77*	1.02
Taian	0.77*	0.87*	0.83*	1.18
Zaozhuang	0.53**	0.57*	0.53**	0.61
Zibo	0.80*	0.85*	0.80*	1.15
Dongying	0.58**	0.64*	0.58**	0.66
Weihai	0.78*	0.81*	0.78*	1.07
Linyi	0.71*	0.78*	0.71*	0.94
Qingdao	0.81*	0.85*	0.81*	1.17
Binzhou	0.72*	0.79*	0.72*	0.96
Rizhao	0.52**	0.57**	0.52**	0.60
Yantai	0.83*	0.87*	0.83*	1.24
Weifang	0.63*	0.67*	0.63**	0.76

**: *P* < 0.01, *: *P* < 0.05.

### Modelling the effects of sociodemographic factors on the associations between internet query and pertussis infections

**Fig. 4.**
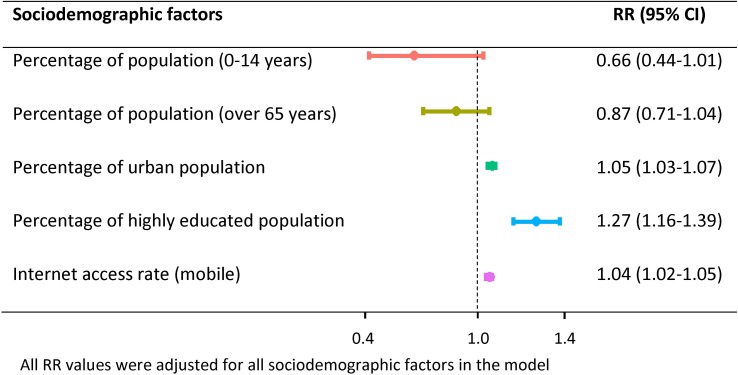
The RR of CTRM associated with sociodemographic factors using GLM in Shandong province, 2009–2017.

The GLM model showed that the CTRM was significantly positively associated with the percentage of urban population, the proportion of highly educated population and the internet access rate (cell phone) (Fig. 4). The RR of CTRM associated with the percentage of urban population was 1.05 (95% CI 1.03–1.07), and the proportion of highly educated population had the highest RR of 1.27 (95% CI 1.16–1.39). Furthermore, the internet access rate (cell phone) was positively associated with the CTRM with a RR of 1.04 (95% CI 1.02–1.05). As these sociodemographic factors were significantly associated with the CTRM, these three factors were identified for further analysis.

### Stratified effects of sociodemographic factors on the association

**Fig. 5.**
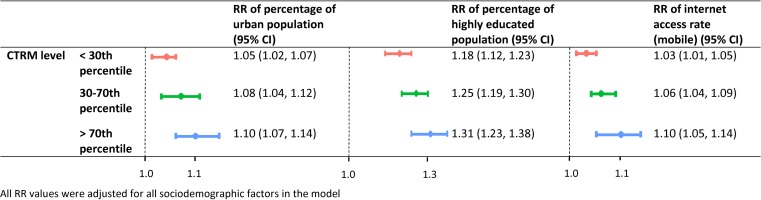
The stratified RRs of CTRM associated with identified sociodemographic factors in the CTRM with GLMs in Shandong province, 2009–2017.

As indicated in Figure 5, there were increasing trends in the RRs of CTRM associated with each identified sociodemographic factor when we stratified the CTRM. In the areas with the highest CTRM (>70th percentile), the RRs of percentage of urban population, proportion of highly educated population and internet access rate (cell phone) were 1.10 (95% CI 1.07–1.14), 1.31 (95% CI 1.23–1.38) and 1.10 (95% CI 1.05–1.14), respectively. However, the RRs of those in the regions with the lowest CTRM (<30th percentile) were 1.05 (95% CI 1.02–1.07), 1.18 (95% CI 1.12–1.23) and 1.03 (95% CI 1.01–1.05), respectively.

### Regression tree analysis

**Fig. 6.**
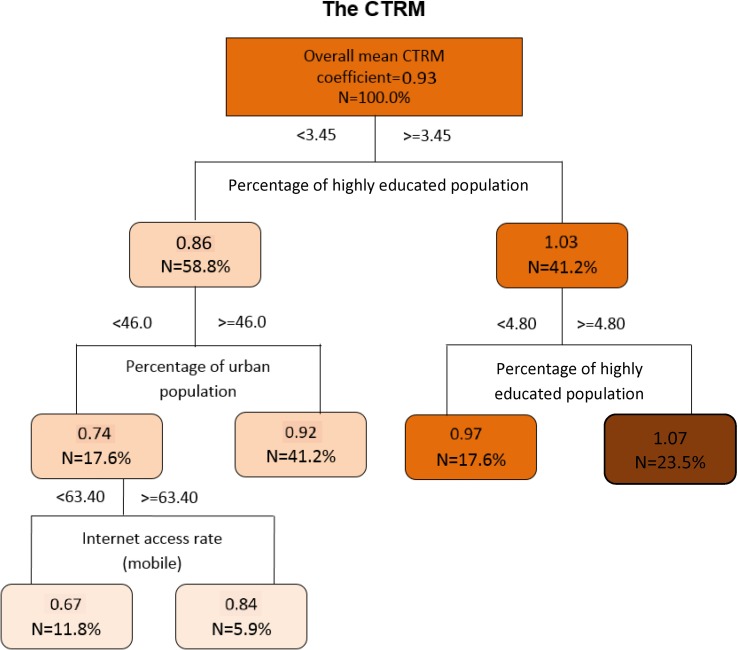
The regression tree modelling the hierarchical relationship between CTRM of pertussis infections and internet query with sociodemographic factors in Shandong province between 2009 and 2017 (the regression trees showed the threshold values and mean correlation coefficient; *N* is the percentage of entire data in the cell (the number of cities)).

Based on the regression trees portrayed in Supplementary Figure S2 and Figure 6, the percentage of the highly educated population was the first splitting variable in all models. That is, the highly educated rate determined to be the most important factor affecting the variation in the correlations between pertussis infections and BI. The mean correlation coefficient between time-series data of weekly pertussis notifications and that of the BI rose from 0.69 to 0.80 when the percentage of highly educated population was ≥4.80 (Supplementary Fig. S2A). When the percentage of highly educated population was ≥3.45, and the internet access rate (cell phone) was ≥79.0, the mean correlation coefficient between peak number of weekly pertussis notifications and that of the BI increased from 0.75 to 0.86 (Supplementary Fig. S2B). Moreover, the mean correlation coefficient between increasing intensity index of pertussis notifications and that of the BI increased from 0.70 to 0.81 when the percentage of highly educated population was ≥4.80 (Supplementary Fig. S2C). Similarly, the mean CTRM value climbed from 0.93 to 1.07 for the same change in increasing intensity (Fig. 6).

## Discussion

To the best of our knowledge, this is the first attempt to examine the effects of sociodemographic factors on internet query in disease monitoring in China. The results showed that, in general, there were significant correlations between pertussis infections and internet query data in Shandong province, as well as substantial spatial variation. Moreover, we found that sociodemographic factors, including the percentage of urban population, the percentage of highly educated population and the internet access rate (cell phone) can affect such correlations. This study may provide important new insights on internet query-based surveillance to better understand the predictive value of this new tool by varying sociodemographic factors.

The study found that internet query had the strongest correlation with pertussis activity (the highest CTRM value) in Yantai, Qingdao and Taian, followed by Zibo, Jinan and Weihai. The result indicated that internet query in those areas can better track the variation, the size of outbreak and the spread speed of transmission of pertussis.

The results showed that percentages of urban or highly educated population as well as internet access rate (cell phone) were positively associated with the relationship between temporal risk indices of pertussis infections and that of internet query (the CTRM). Moreover, the RRs associated with identified sociodemographic factors were stratified by the CTRM, with higher RRs observed when larger CTRM values were considered.

Internet query-based surveillance relies on the premise that people who contract a disease will actively seek information about their condition from the internet and that disease activity can be estimated by tracking changes in frequencies of related internet searches for key terms [25]. The regions with a high percentage of urban or highly educated population likely as a population to have more knowledge about health and disease [28]. Moreover, the high internet access rate means the people can more easily search disease-related information from the internet. These may lead to higher correlation coefficients between pertussis infections and BI in the study, which indicates a better monitoring of pertussis infections using internet query data.

The regression tree models confirmed that the percentage of the highly educated population played a key role in the occurrence of strong correlations between pertussis infections and internet query. However, the percentage of urban population and the internet access rate differentially contributed to the models. The results showed that strong correlations and larger CTRM, which refer to better pertussis monitoring using internet query data, in general, can be observed in the areas with higher percentage of highly educated population, higher proportion of urban population and higher internet access rate.

The study suggested that public health authorities should specifically assess the sociodemographic conditions, and then develop location-specific prediction models using internet query data. Our findings are important to public health officers and government, as this study provided potential threshold values of identified sociodemographic factors linking the association of official pertussis surveillance data with internet query data. The values may assist the identification of regions in which there is a better pertussis monitoring using internet query data. Moreover, we suggested that the models may be improved if more predictors, such as climate factors can be included to better forecast pertussis epidemics.

Some limitations of the study should be acknowledged. First, CNNDRS only includes the reported number of cases, and this database does not include patients who have pertussis but do not seek medical care or have been misdiagnosed by a clinic and laboratory. In particular this may be occurring in locations that are less urbanised, less highly educated and have lower rates of mobile internet access. Relatedly, different internet-seeking behaviours between different communities can adversely influence the accuracy of internet-based surveillance [41]. Finally, the model of this study may be improved if more sociodemographic factors can be included, such as healthcare access, gender and age. Improved understanding of how such sociodemographic factors influence the use of the internet in queries about disease could contribute to reducing many limitations of search query data in the future.

## Conclusions

In conclusion, the study suggested that internet query analysis has considerable potential in monitoring pertussis infections in Shandong province but with spatial differences in its utility, and that urbanisation, education and mobile internet access were positively correlated with the association between pertussis notifications and internet query data. The results may be significant for developing location-specific prediction models using internet query data, providing a foundation for constructing early warning systems using such data.

## Supplementary material

For supplementary material accompanying this paper visit https://doi.org/10.1017/S0950268819001924. click here to view supplementary material

## References

[1] World Health Organization. (2018) WHO vaccine-preventable diseases: monitoring system. 2018 global summary (http://apps.who.int/immunization_monitoring/globalsummary/countries?countrycriteria%5Bcountry%5D%5B%5D=CHN&commit=OK). Accessed 8 May 2019.

[2] Schmidtke AJ (2012) Population diversity among *Bordetella pertussis* isolates, United States, 1935–2009. Emerging Infectious Diseases 18, 1248. 22841154 10.3201/eid1808.120082 PMC3414039

[3] Kamiya H (2012) Transmission of *Bordetella holmesii* during pertussis outbreak, Japan. Emerging Infectious Diseases 18, 1166. 22709586 10.3201/eid1807.120130 PMC3376812

[4] Octavia S (2012) Newly emerging clones of *Bordetella pertussis* carrying prn2 and ptxP3 alleles implicated in Australian pertussis epidemic in 2008–2010. Journal of Infectious Diseases 205, 1220–1224. 22416243 10.1093/infdis/jis178

[5] Zeng Q (2016) Time series analysis of temporal trends in the pertussis incidence in Mainland China from 2005 to 2016. Scientific Reports 6, 32367. 27577101 10.1038/srep32367 PMC5006025

[6] WHO (2018) Pertussis (https://www.who.int/immunization/monitoring_surveillance/burden/vpd/surveillance_type/passive/pertussis/en/). Accessed 8 May 2019.

[7] WHO (2015) Weekly epidemiological record (https://www.who.int/wer/2015/wer9035.pdf?ua=1). Accessed 8 May 2019.

[8] Huang H (2015) Epidemiological features of pertussis resurgence based on community populations with high vaccination coverage in China. Epidemiology & Infection 143, 1950–1956. 25286969 10.1017/S095026881400260X PMC9507247

[9] Guo B (2013) Systematic review of reporting rates of adverse events following immunization: an international comparison of post-marketing surveillance programs with reference to China. Vaccine 31, 603–617. 23200940 10.1016/j.vaccine.2012.11.051

[10] Gambhir M (2015) A change in vaccine efficacy and duration of protection explains recent rises in pertussis incidence in the United States. PLoS Computational Biology 11, e1004138. 25906150 10.1371/journal.pcbi.1004138 PMC4408109

[11] Zhang Q (2013) High-resolution melting analysis for the detection of two erythromycin-resistant *Bordetella pertussis* strains carried by healthy schoolchildren in China. Clinical Microbiology and Infection 19, E260–E262. 23480481 10.1111/1469-0691.12161

[12] Wang Z (2014) High prevalence of erythromycin-resistant *Bordetella pertussis* in Xi'an, China. Clinical Microbiology and Infection 20, O825–O830. 24816168 10.1111/1469-0691.12671

[13] Chan EH (2010) Global capacity for emerging infectious disease detection. Proceedings of the National Academy of Sciences 107, 21701–21706. 10.1073/pnas.1006219107 PMC3003006 21115835

[14] Yuan Q (2013) Monitoring influenza epidemics in China with search query from Baidu. PLoS One 8, e64323. 23750192 10.1371/journal.pone.0064323 PMC3667820

[15] Wang L (2008) Emergence and control of infectious diseases in China. Lancet 372, 1598–1605. 18930534 10.1016/S0140-6736(08)61365-3 PMC7138027

[16] Project TS (2011) Assessment of syndromic surveillance in Europe. Lancet 378, 1833–1834. 22118433 10.1016/S0140-6736(11)60834-9

[17] Kang M (2013) Using google trends for influenza surveillance in South China. PLoS One 8, e55205. 23372837 10.1371/journal.pone.0055205 PMC3555864

[18] Cho S (2013) Correlation between national influenza surveillance data and google trends in South Korea. PLoS One 8, e81422. 24339927 10.1371/journal.pone.0081422 PMC3855287

[19] Shin S-Y (2016) Correlation between national influenza surveillance data and search queries from mobile devices and desktops in South Korea. PLoS One 11, e0158539. 27391028 10.1371/journal.pone.0158539 PMC4938422

[20] Seo D-W (2014) Cumulative query method for influenza surveillance using search engine data. Journal of Medical Internet Research 16, e289. 25517353 10.2196/jmir.3680 PMC4275481

[21] Polgreen PM (2008) Using internet searches for influenza surveillance. Clinical Infectious Diseases 47, 1443–1448. 18954267 10.1086/593098

[22] Milinovich GJ, Magalhães RJS and Hu W (2015) Role of big data in the early detection of Ebola and other emerging infectious diseases. Lancet Global Health 3, e20–e21. 25539964 10.1016/S2214-109X(14)70356-0

[23] Li Z (2017) Dengue Baidu Search Index data can improve the prediction of local dengue epidemic: a case study in Guangzhou, China. PLOS Neglected Tropical Diseases 11, e0005354. 28263988 10.1371/journal.pntd.0005354 PMC5354435

[24] Zhang Y (2017) Monitoring pertussis infections using internet search queries. Scientific Reports 7, 10437. 28874880 10.1038/s41598-017-11195-z PMC5585203

[25] Milinovich GJ (2014) Using internet search queries for infectious disease surveillance: screening diseases for suitability. BMC Infectious Diseases 14, 690. 25551277 10.1186/s12879-014-0690-1 PMC4300155

[26] Pollett S (2015) Validating the use of Google trends to enhance pertussis surveillance in California. PLoS Currents 7, 1–10. 10.1371/currents.outbreaks.7119696b3e7523faa4543faac87c56c2 PMC4626035 26543674

[27] Phillips CA (2018) Relationship between state-level Google online search volume and cancer incidence in the United States: retrospective study. Journal of Medical Internet Research 20, e6. 29311051 10.2196/jmir.8870 PMC5778251

[28] Pollett S (2016) Evaluating Google Flu Trends in Latin America: important lessons for the next phase of digital disease detection. Clinical Infectious Diseases 64(1), 34–41. 27678084 10.1093/cid/ciw657 PMC6394128

[29] Butler D (2013) When Google got flu wrong. Nature 494, 155. 23407515 10.1038/494155a

[30] Nsoesie EO (2016) Social media as a sentinel for disease surveillance: what does sociodemographic status have to do with it? PLoS Currents 8, e1. 10.1371/currents.outbreaks.cc09a42586e16dc7dd62813b7ee5d6b6 PMC5222536 28123858

[31] Henly S (2017) Disparities in digital reporting of illness: a demographic and socioeconomic assessment. Preventive Medicine 101, 18–22. 28528170 10.1016/j.ypmed.2017.05.009 PMC5553633

[32] Medaglia R and Zhu D (2017) Public deliberation on government-managed social media: a study on Weibo users in China. Government Information Quarterly 34, 533–544.

[33] Statcounter (2018) Search engine market share China (http://gs.statcounter.com/search-engine-market-share/all/china). Accessed 8 May 2019.

[34] National Statistics Bureau of China (2010) The sixth national population census data (http://data.stats.gov.cn/). Accessed 8 May 2019.

[35] Xu L (2010) Socio-economic factors affecting the success of tuberculosis treatment in six counties of Shandong Province, China. The International Journal of Tuberculosis and Lung Disease 14, 440–446. 20202302

[36] Wang Y (2018) Time series modeling of pertussis incidence in China from 2004 to 2018 with a novel wavelet based SARIMA-NAR hybrid model. PLOS ONE 13, e0208404. 30586416 10.1371/journal.pone.0208404 PMC6306235

[37] National Health Commission of the PRC (2007) Pertussis diagnostic criteria (http://www.nhfpc.gov.cn/zwgkzt/s9491/201410/52040bc16d3b4eecae56ec28b3358666.shtml). Accessed 8 May 2019.

[38] Pervaiz F (2012) Flubreaks: early epidemic detection from Google flu trends. Journal of Medical Internet Research 14, e125. 23037553 10.2196/jmir.2102 PMC3510767

[39] Milinovich GJ (2014) Internet-based surveillance systems for monitoring emerging infectious diseases. The Lancet Infectious Diseases 14, 160–168. 24290841 10.1016/S1473-3099(13)70244-5 PMC7185571

[40] Dunn CE (2001) Analysing spatially referenced public health data: a comparison of three methodological approaches. Health & Place 7, 1–12. 11165151 10.1016/s1353-8292(00)00033-2

[41] Zhang Y (2018) Using Google Trends and ambient temperature to predict seasonal influenza outbreaks. Environment International 117, 284–291. 29778013 10.1016/j.envint.2018.05.016

[42] Wen T-H (2006) Spatial mapping of temporal risk characteristics to improve environmental health risk identification: a case study of a dengue epidemic in Taiwan. Science of the Total Environment 367, 631–640. 16584757 10.1016/j.scitotenv.2006.02.009

[43] Fisher RA (1921) On the ‘probable error’ of a coefficient of correlation deduced from a small sample. Metron 1, 1–32.

[44] Weiss S (2016) Correlation detection strategies in microbial data sets vary widely in sensitivity and precision. The ISME Journal 10, 1669. 26905627 10.1038/ismej.2015.235 PMC4918442

[45] Wu J (2015) Buruli ulcer disease and its association with land cover in southwestern Ghana. PLoS Neglected Tropical Diseases 9, e0003840. 26091265 10.1371/journal.pntd.0003840 PMC4474842

[46] Wang P, Goggins WB and Chan EY (2018) Associations of Salmonella hospitalizations with ambient temperature, humidity and rainfall in Hong Kong. Environment International 120, 223–230. 30103121 10.1016/j.envint.2018.08.014

[47] Wu J and Jackson L (2017) Inverse relationship between urban green space and childhood autism in California elementary school districts. Environment International 107, 140–146. 28735150 10.1016/j.envint.2017.07.010 PMC6104398

[48] Liu K (2016) Using Baidu search index to predict Dengue outbreak in China. Scientific Reports 6, 38040. 27905501 10.1038/srep38040 PMC5131307

[49] Breiman L (2017) Classification and Regression Trees. Abingdon, United Kingdom: Routledge.

