# Supplementary material for: Association of sociodemographic factors and internet query data with pertussis infections in Shandong, China
Source: Epidemiol Infect. 2019 Nov 15;147:e302. doi: 10.1017/S0950268819001924 (PMC6873159; doi:10.1017/S0950268819001924)
Supplement: Supplementary file 1 [file S0950268819001924sup001.docx]

# **Supplementary information**

# **Association of sociodemographic factors and internet query data with pertussis infections in Shandong, China**

**Table S1. The search queries used in data analysis.**

| Pertussis | Pertussis infections |
| --- | --- |
| Pertussis vaccine | Pertussis prevention |
| Pertussis treatments | Pertussis infection period |
| Pertussis symptoms | Pertussis mortality |
| Pertussis medications | Pertussis in children |

**Figure S1. City-specific percentage of urban population, GPC, percentage of highly educated population and** **percentages of population (0-14 years old, 15-64 years old and over 65 years old) in Shandong.**


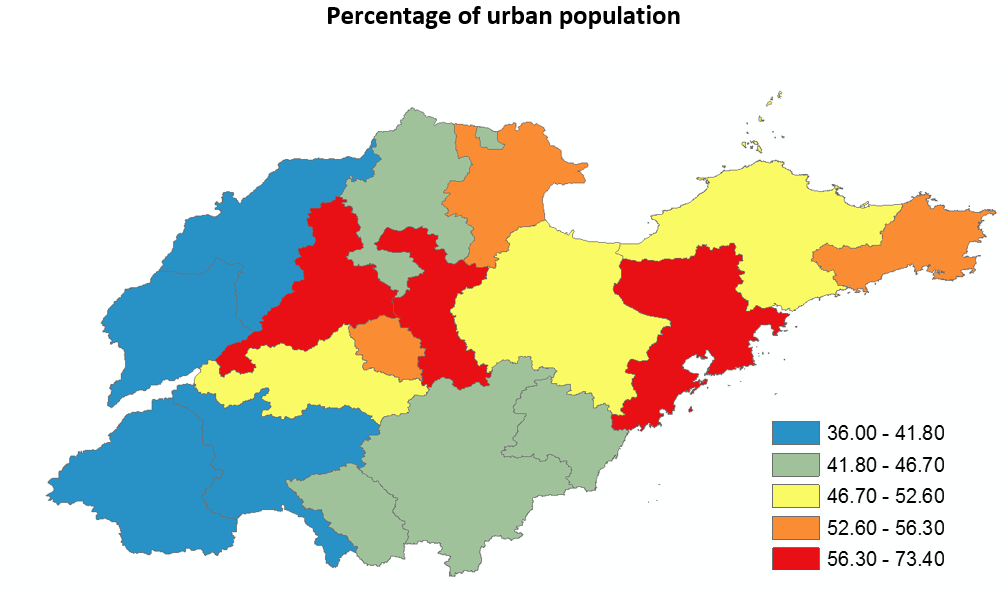


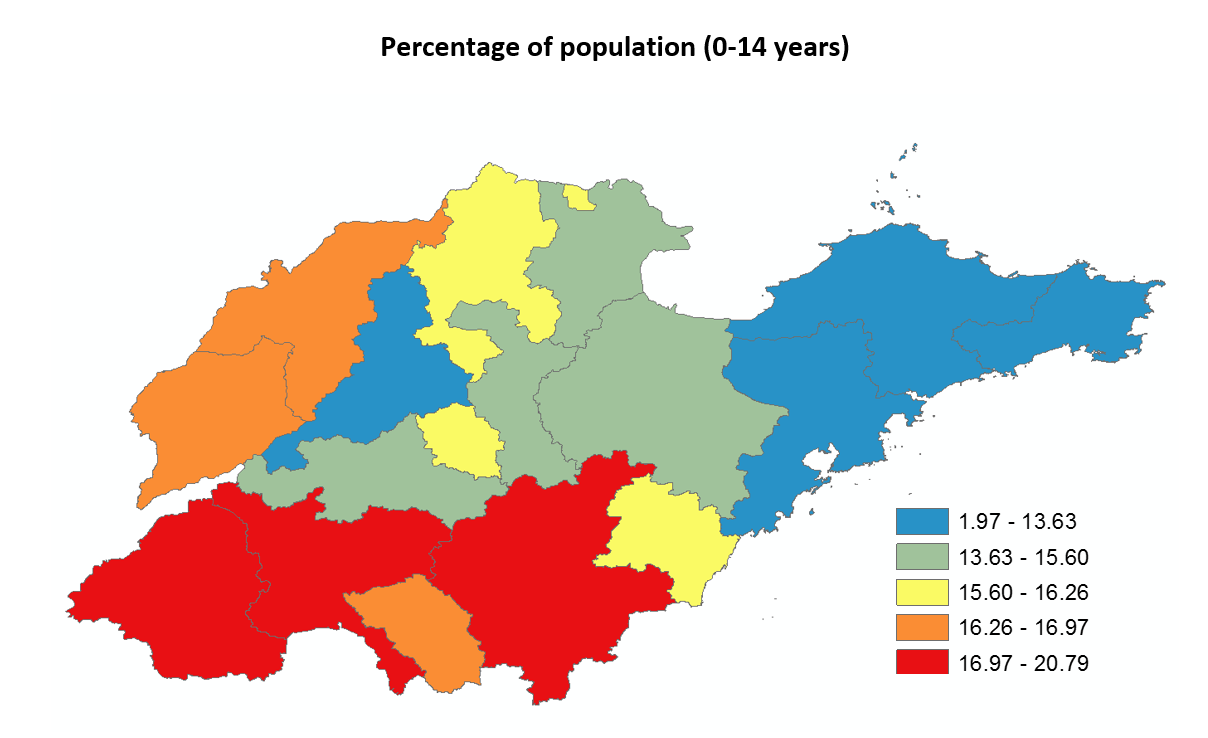


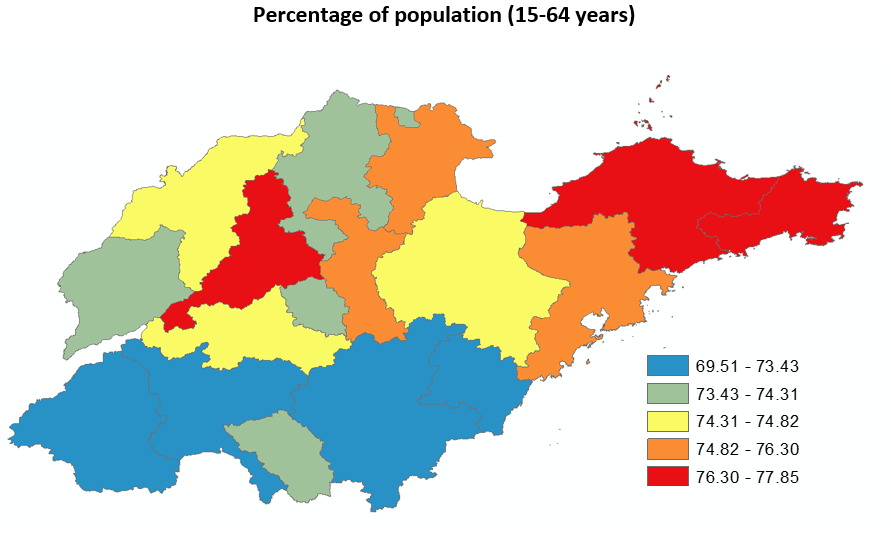


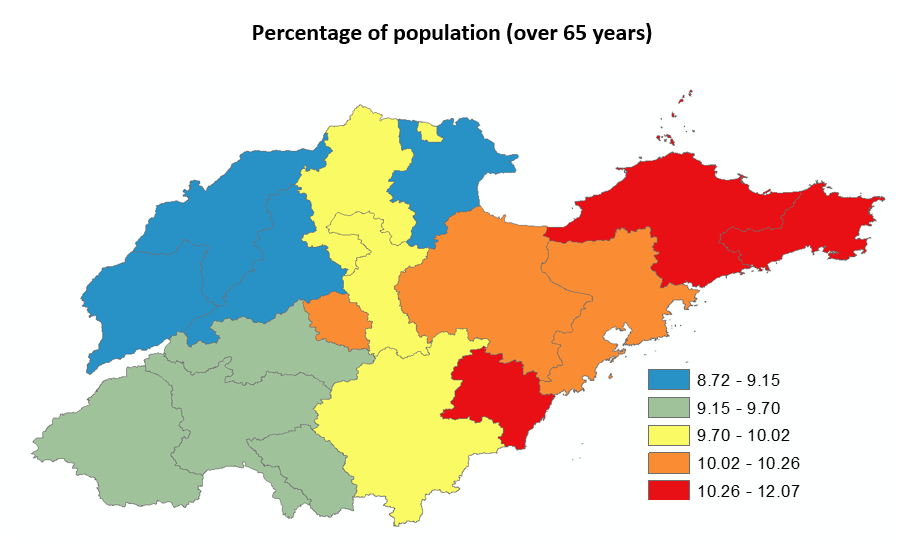

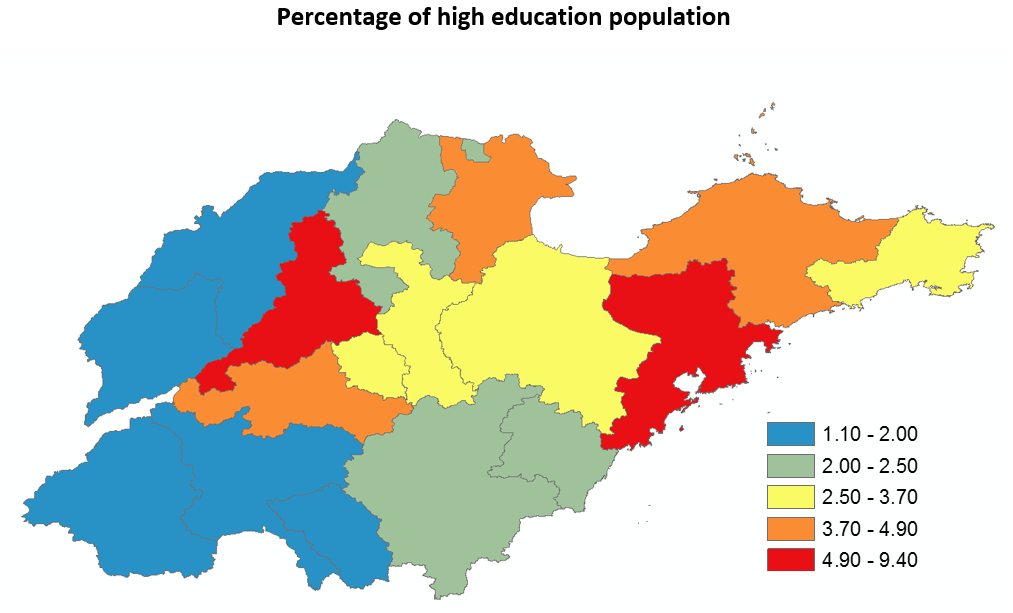

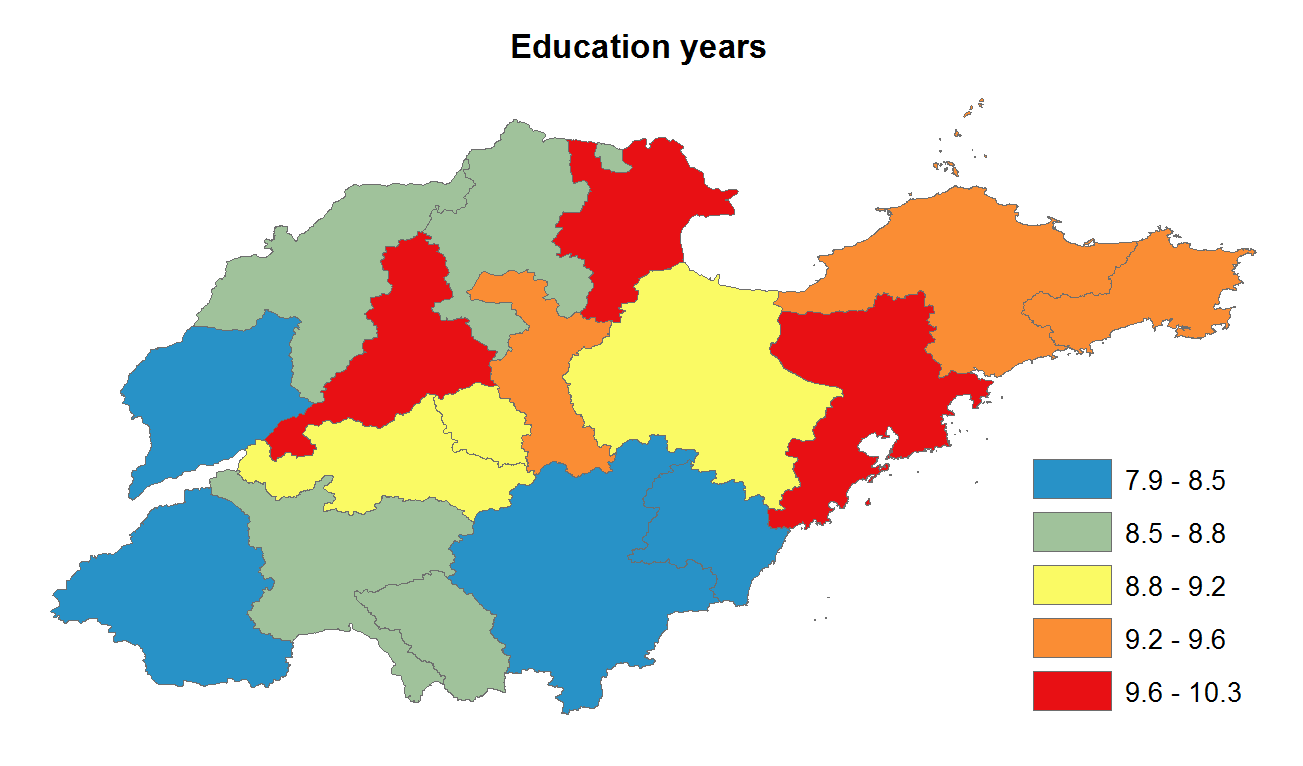

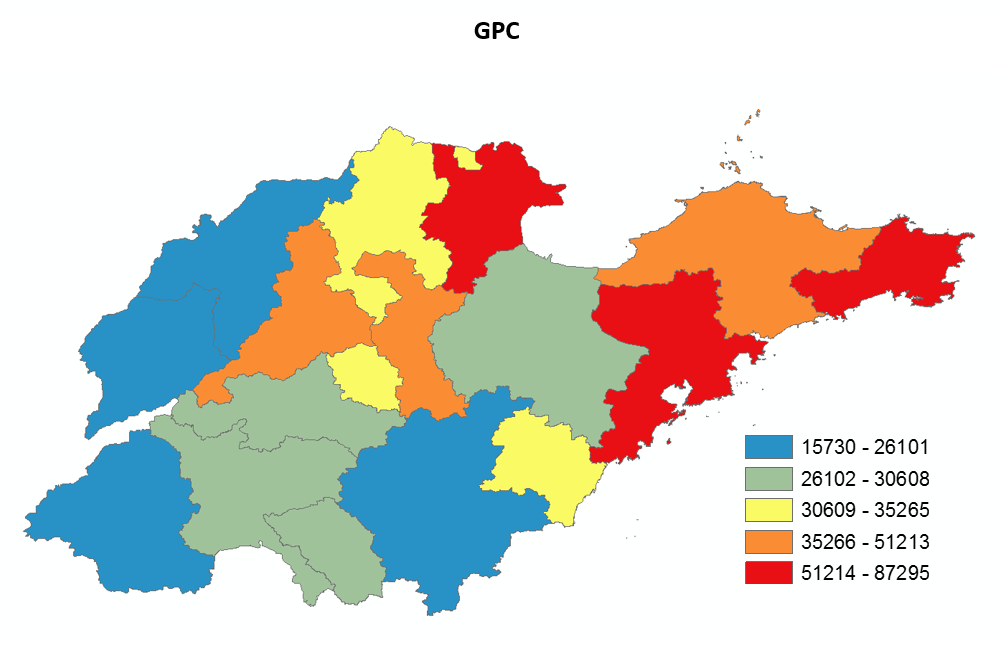

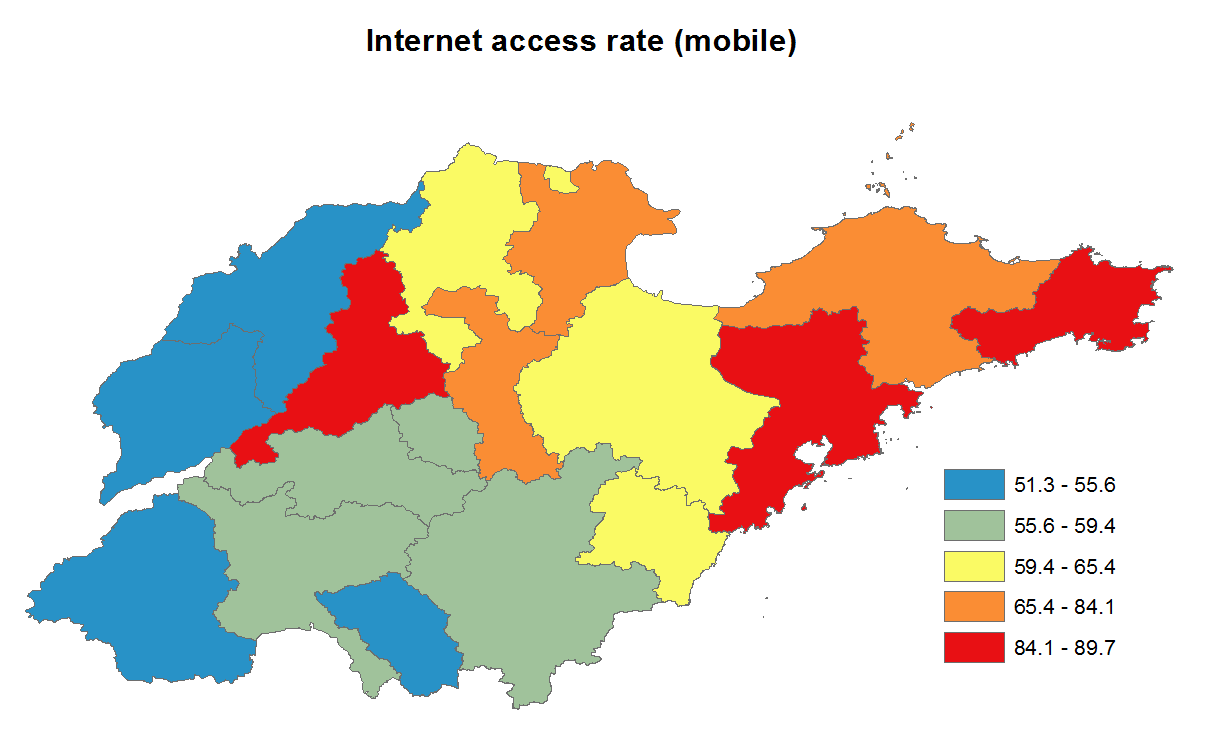


**Percentage of highly educated population**


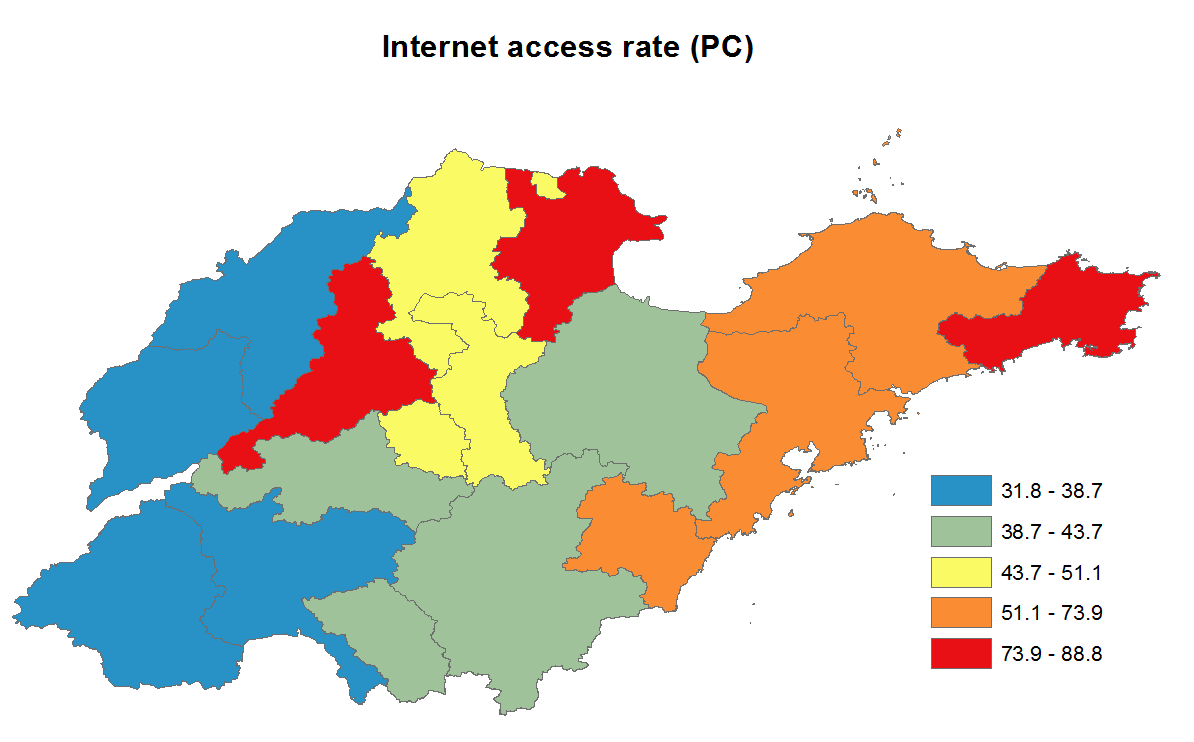


**Figure S2. The regression tree modelling the hierarchical relationship between the temporal risks (time-series data, peaking number and increasing intensity) of pertussis infections and internet query with sociodemographic factors in Shandong province between 2009 and 2017** (The regression trees showed the threshold values, mean correlation coefficient; N is the percentage of entire data in the cell (the number of cities))**.**

**A**


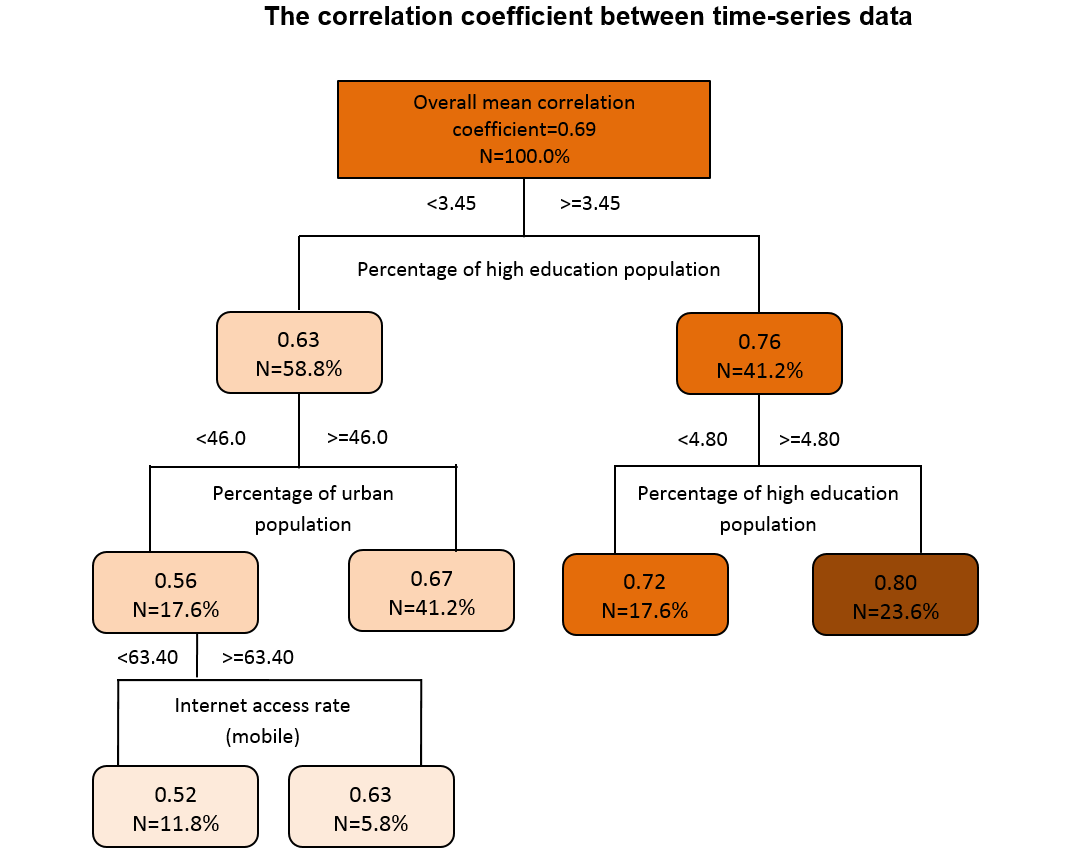


Percentage of highly educated population

Percentage of highly educated population


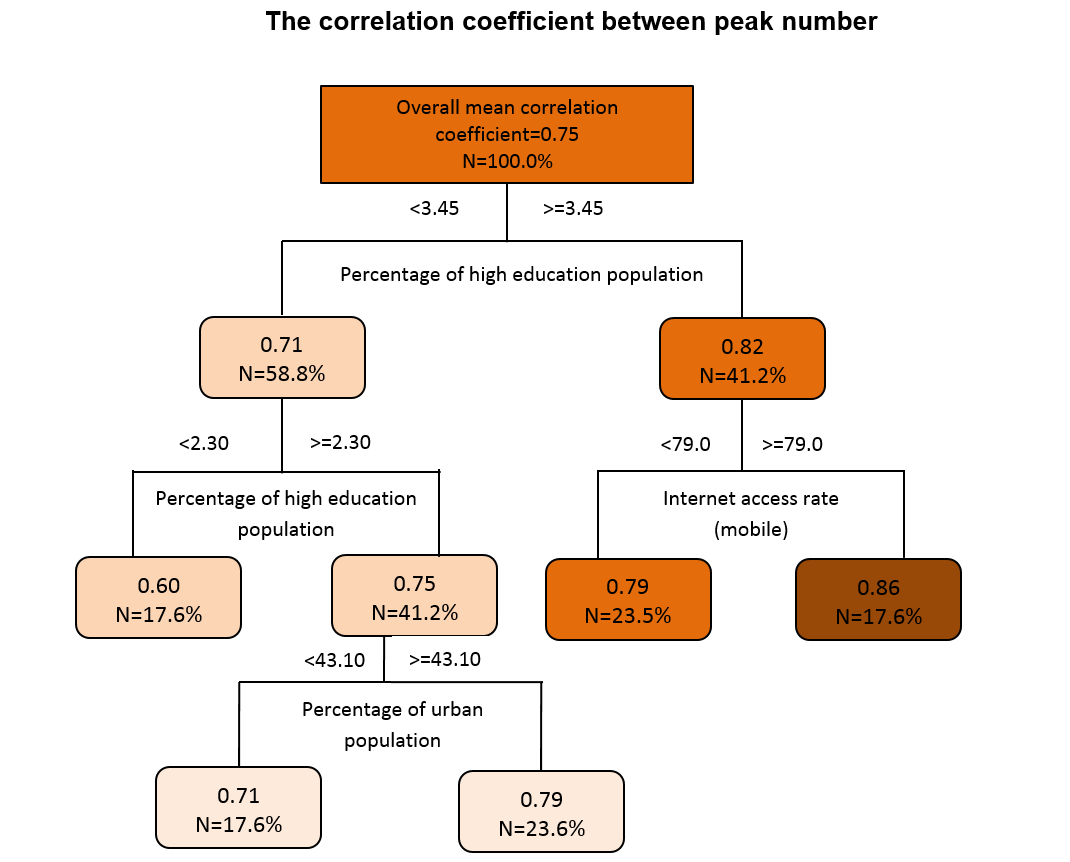


Percentage of highly educated population

Percentage of highly educated population

**B**


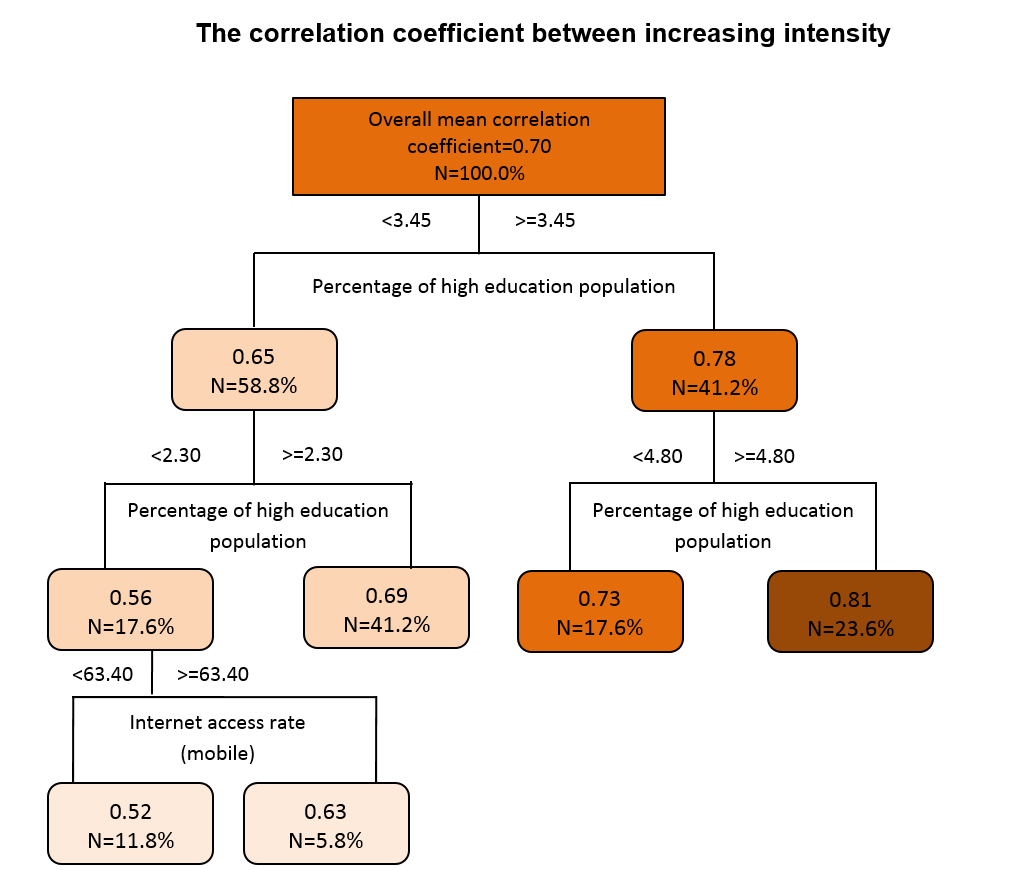


Percentage of highly educated population

Percentage of highly educated population

Percentage of highly educated population

**C**
